# Prevalence of Hospital-Acquired Enterococci Infections in Two Primary-Care Hospitals in Osogbo, Southwestern Nigeria

**DOI:** 10.4314/ajid.v5i2.66513

**Published:** 2011

**Authors:** Kafayat Olayinka Olawale, Solomon Olufemi Fadiora, Samuel Sunday Taiwo

**Affiliations:** Department of Medical Microbiology/ Parasitology and ^2^Surgery, College of Health Sciences, Ladoke Akintola University of Technology, PMB 4400, Osogbo, Nigeria

**Keywords:** Nosocomial, Prevalence, Enterococcus, Vancomycin-Resistance, Primary Care

## Abstract

Enterococci are opportunistic bacteria that become pathogenic when they colonize niches where they are not normally found. Of recent, they have become major cause of nosocomial infections, especially of the bloodstream, urinary tract and surgical sites. The aim of this study is to determine the point-prevalence rate of human enterococci infections among hospitalized patients in Osogbo, Nigeria. The study was conducted between January and June 2009 in two primary-care hospitals in Osogbo and involved a total of 118 patients who developed clinical evidence of infection at least 48 hours after hospital admission. Appropriate clinical samples were collected from the patients after an informed consent and cultured for isolation/biochemical identification of *Enterococcus* species at the Bacteriology Laboratory of Ladoke Akintola University of Technology, Osogbo using standard microbiological methods. There were 525 hospital admissions within the time frame of the study of which 118 (22.5%) developed hospital acquired infection (HAI); 58 (49.2%) of which cultured positive for bacterial pathogens. Enterococci were isolated from infective focus in 7 patients, giving a prevalence rate of hospital-acquired enterococci infection of 5.9%. Two species of *Enterococcus* were identified; *Enterococcus faecalis* from urinary tract infection (UTI) and surgical site infection (SSI) of 6 (85.7%) patients and *Enterococcus faecium* from UTI in 1 (14.3%) patient. Other bacteria recovered from other infective foci were *Klebsiella* spp 31.0%, *Pseudomonas* spp 20.7%, *Staphylococcus aureus* 17.2%, *Escherichia coli* 12.1%, *Staphylococcus epidermidis* 3.4%, *Streptococcus pneumoniae* 1.7% and *Serratia* spp 1.7%. All the enterococci isolates were multiply antibiotic resistant, and 42.9% were vancomycin-resistant enterococci (VRE) with the VRE strains showing resistance to wider range of antibiotics than the vancomycin-sensitive strains. Other Gram-positive and Gram negative bacterial isolates also demonstrated multiple resistance to all commonly available antibiotics in this community except *E. coli* and *Pseudomonas* spp which were relatively sensitive to ciprofloxacin and ceftazidime. This limited study demonstrated a high prevalence rate of multiple antibiotic resistant enterococci infections among hospitalized patients in this environment. There is need for systematic surveillance of hospitals for enterococci infections; prudent use and rational prescription of antibiotics and stringent measures to reduce the prevalence rate by health education on infection control measures such as isolation, cleaning, disinfection and sterilization.

## Introduction

Enterococci are hardy, facultatively anaerobic Gram positive cocci in pairs or short chains that can grow and survive in many environments (Murray, 1990). They are part of normal flora of intestine of humans and animals but may be responsible for serious infections. Of the over 20 *Enterococcus* species (Facklam *et al*, 2002), 2 species are particularly pathogenic to man; *Enterococcus faecalis* causes 85–90% of enterococci infections while *Enterococcus faecium* causes 5–10% (Lewis & Zervos, 1990; Gordon *et al*, 1992; Patterson *et al*, 1995). Other *Enterococcus* species known to cause human infections include *E. avium, E. gallinarium, E. casseliflavus, E. dirans, E. raffinosus* and *E. mundtii*

Enterococci are among the most frequent causes of nosocomial infection, particularly in intensive care units (ICU) where they are selected by therapy with cephalosporin and other antibiotics to which they are resistant. They are transmitted from person to person primarily on the hand of hospital personnel, some of whom may carry the organism in their gastrointestinal tracts. Occasionally, enterococci are transmitted on medical devices. In patients, the most common sites of enterococci infections are the urinary tract, wound and biliary tract along with other species of bacteria where it may be difficult to define the pathogenic role of the enterococci (Murray, 1990). In neonates, meningitis and bacteraemia may occur and endocarditis may occur in adults. Enterococci infection is equally distributed between sexes (Gordon *et al*, 1992), although urinary tract infections are more common in healthy women than men and in elderly patients due to high incidence of urinary instrumentation.

In the routine microbiology laboratory, enterococci are characterized by their morphologic appearance on Gram stain and on culture, and are distinguished from the non-group D streptococci by their ability to survive in the presence of 40% bile, ability to hydrolyze aesculin, growth in 6.5% NaCl and a positive pyrrolidonylarylamidase test (Facklam *et al*, 2002). The treatment of enterococci infection is usually problematic because they are usually resistant to β-lactam antibiotics and aminoglycosides, though synergistic action of a combination of these drugs may be effective. The glycopeptide, vancomycin was the drug of choice but resistance to this drug is now on the increase. Newer antibiotics, such as the combination of quinupristin and dalfopristin are currently used to treat vancomycin resistant enterococci infection (Arias & Murray, 2008).

In Nigeria, the role of enterococci in clinical infections has not been appreciated hence reports of enterococci infections are very few. A previous study in Ilorin (Taiwo *et al*, 2002) reported 2.8% of 642 bacteria wound isolates to be *Streptococcus* (*Enterococcus*) *faecalis*. Another study in Lagos reported that 11% of 35 vancomycin susceptible *E. faecalis* isolates obtained from different clinical specimens exhibited high-level resistance (HLR) to gentamicin (MIC ≥ 2,000 µg/ml) and 32% exhibited HLR to streptomycin (Iregbu *et al*, 2002). In recent times in the teaching hospital in Osogbo, we have isolated *E. faecalis* from blood stream and wound infections (Taiwo *et al*, 2008; Fadiora *et al*, 2009) amongst other pathogens, however, the prevalence and magnitude of enterococci infections in this environment is largely unknown. The objectives of this study are; to determine the prevalence of hospital acquired enterococci infection and the common *Enterococcus* species in primary-care hospitals in Osogbo, Nigeria and to determine their susceptibility profile to commonly prescribed antibiotics. This information is to serve as a pilot data for more extensive molecular epidemiology of enterococci infections that will be necessary for the formulation of control policy in this environment.

## Materials and Method

### Study area

This research is a descriptive cross sectional study carried out over a 6 month period (January to June 2009) in two primary-care hospitals and the bacteriology laboratory of the Ladoke Akintola University of Technology, Osogbo Southwestern Nigeria. The two hospitals offer general medical and surgical as well as gynaecologic and obstetric services, and were selected because of their relatively high patient patronage and consent of their hospital management board.

### Subjects

The subjects were patients who developed clinical evidence of infections at least 48 hours after hospital admission. One hundred and eighteen patients were studied. Informed consent was obtained from each subject and the ethical approval of the two hospital management was obtained before the conduct of the study. Demographic and clinical data were collected from each patient into a designed form.

### Laboratory procedure

Appropriate clinical specimens (blood, urine, wound swabs, sputum and stool) were collected from each subject as applicable and transported to the bacteriology laboratory for processing, aerobic cultures and isolation on Sheep Blood agar/other appropriate culture media, and biochemical identification of enterococci and other bacterial pathogens according to recommended techniques and procedures (Cheesbrough, 2000).

#### Isolation/identification of enterococci

Enterococci were identified on Sheep Blood agar plate as non-haemolytic 0.5–1mm size streptococci-like colonies; on MacConkey agar as small dark-red magenta colonies and on CLED agar as small yellow colonies from fermentation of lactose (Cheesbrough, 2000). The colonies were confirmed as enterococci with Gram stain positivity, negative catalase test, positive bile-aesculin (bile insolubility) test, growth in 6.5% NaCl broth and as *Enterococcus* species by specific sugar (glucose, lactose, mannitol, sorbitol and arabinose) fermentation reactions (Facklam & Collins, 1989; Facklam *et al*, 1989).

#### Antibiotic susceptibility test

The susceptibility of each enterococci isolate to oxacillin and vancomycin was determined using Clinical and Laboratory Standards Institute (CLSI) disk diffusion method (CLSI, 2007) on Mueller-Hinton agar (supplemented with 2% NaCl) with 1µg oxacillin and 30µg vancomycin disks and incubating at 35°C for 24 hours. Oxacillin zone diameter (ZD) of inhibition ≥14mm defined oxacillin susceptibility in enterococci while vancomycin ZD ≥17mm defined vancomycin susceptibility (CLSI, 2007).

Susceptibility of each isolate to other antibiotics (ampicillin 10µg, erythromycin 15µg, gentamicin 10µg, cotrimoxazole 25µg, tetracycline 10µg, ceftazidime 30µg and ciprofloxacin 5µg) was performed using the disk diffusion method of Bauer *et al* (1966). ZD for susceptibility to these antibiotics in enterococci were; ampicillin ≥17mm, erythromycin ≥23mm, gentamicin ≥15mm, cotrimoxazole ≥16mm, tetracycline ≥19mm, ceftazidime ≥18mm and ciprofloxacin ≥21mm (CLSI, 2007). *Staphylococcus aureus* ATCC 25923 serve as negative and *E. faecalis* ATCC 51299 as positive control strains.

### Data entry and Statistical analysis

All data (demographic and clinical) were entered into Window Vista 2007 laptop computer with GraphPad statistical software. Frequency tables were generated and relationship between variables tested with Chi square or Fisher's Exact test with significant value set at *P*<0.05.

## Results

Over the 6 months period of study, there were 525 hospital admissions in the two primary-care hospitals in Osogbo, Nigeria. A total of 118 patients who developed clinical evidence of infection 48 hours after hospitalization were enrolled into the study. The hospital-acquired infection (HAI) rate in the two hospitals was 22.5%. Table 2 show the age and sex distribution of the eligible patients. The age group 20–29years constitute the largest proportion (42.4%) followed by age group 30–39 years (23.7%) and others as shown in Table 2. Table 3 shows the prevalence of enterococci infection of 5.9% (7 of 118) among the patients, with surgical site and urinary tract infections being the most prevalent with 18.8% and 2.6% rates respectively.

**Table 2:** Prevalence of hospital acquired enterococci infection in two primary- care hospitals in Osogbo, Nigeria

Clinical diseases	Specimen	Number of patients (%)	Enterococci
isolates (%)			
Gastro-enteritis	Stool	18 (15.3)	-
Blood stream infection	Blood	20 (16.9)	-
Respiratory tract infection	Sputum	9 (7.6)	-
Surgical site infection	Swab/biopsy	32 (27.1)	6
Urinary tract infection	Urine	39 (33.1)	1
	**Total**	**118 (100)**	**7 (5.9)**

**Table 3:** Enterococci species identified by sugar fermentation reactions in two primary–care hospitals in Osogbo

Species	No of strain	Sugar reaction (% positive)				
		
		Glucose	Lactose	Mannitol	Sorbitol	Arabinose
*E. faecalis*	6	+ (100)	+ (100)	+ (100)	+ (100)	− (0)
*E. faecium*	1	− (0)	+ (100)	+ (100)	− (0)	+ (100)

Of the 118 patients with clinical infection, 58 (49.2%) were cultured positive for bacterial pathogens (with one microbial pathogen isolated in each case) while 60 (50.8%) were bacteriologically sterile. Enterococci were isolated in 7 (5.9%) patients and 2 species were identified using the sugar fermentation test described by Facklam & Collins (1989); *E. faecalis* in 6 and *E. faecium* in 1 (Table 3). Other non-enterococci bacteria recovered were *Staphylococcus aureus* 10 (17.2%), *Staphylococcus epidermidis* 2 (3.4%), *Streptococcus pneumoniae* 1 (1.7%), *Klebsiella* spp 18 (31.0%), *Serratia* spp 1 (1.7%), *Escherichia coli* 7 (12.1%) and *Pseudomonas* spp 12 (20.7%). The six *E. faecalis* were isolated from cases of SSI (5) and UTI (1) while the only *E. faecium* was isolated from a case of UTI (Table 4).

**Table 4:** Bacterial isolates from hospital acquired infections in two primary-care hospitals in Osogbo, Nigeria

Isolate	Number	Percentage
**Gram positive**		
*Enterococcus faecalis*	6	10.4
*Enterococcus faecium*	1	1.7
*Staphylococcus aureus*	10	17.2
*Staphylococcus epidermidis*	2	3.4
*Streptococcus pneumoniae*	1	1.7
**Gram negative**		
*Klebsiella* spp	18	31.0
*Serratia* spp	1	1.7
*Escherichia coli*	7	12.1
*Pseudomonas* spp	12	20.7
	
**Total**	**58**	**100**

Table 5 shows the resistant pattern of the *Enterococcus* species. All isolates were resistant to ampicillin and oxacillin while 3 (42.9%) including the only *E. faecium* were resistant to vancomycin (VRE). The isolates also showed multi-drug resistant patterns with the VRE showing resistant to wider range of antibiotics (7 antibiotics) than the vancomycin sensitive strains (4–5 antibiotics.

**Table 5:** Resistance pattern of hospital-acquired enterococci isolates in Osogbo, Nigeria

No of antibiotic	Antibiotic Resistance Pattern	No of isolate (%)
4	Amp, Oxa, Ceft, Gen	2 (28.6)
5	Amp, Oxa, Ceft, Gen, Cot	2 (28.6)
8	Amp, Oxa, Ceft, Gen, Cot, Cip, Ery, Van	*3 (42.8)
	**Total**	**7 (100)**

Amp=ampicillin, Oxa=oxacillin, Gen=Gentamicin, Ceft=Ceftazidime, Cot=Cotrimoxazole, Cip=Ciprofloxacin, Ery=Erythromycin, Van=Vancomycin

* include the only *E. faecium* isolated

Table 6 shows the susceptibility profile of other Gram-positive pathogens isolated. Most of the isolates were resistant to commonly used antibiotics in this environment. Table 7 shows antibiotic susceptibility of Gram-negative pathogens isolated in the study. Most of the isolates were also resistant to all antibiotics except *E. coli* which was sensitive to ciprofloxacin (85.7%) and *Pseudomonas* spp which was sensitive to ceftazidime (100%) and ciprofloxacin (58.3%).

**Table 6:** % Resistance of other Gram positive bacteria isolates in two primary-care hospitals in Osogbo

Isolate	Antibiotics (%)							
	
	Amp	Oxa	Gen	Van	Ery	Cot	Ceft	Cip
*S. aureus* (n=10)	10 (100)	5 (50)	10 (100)	2 (20)	6 (60)	10 (100)	10 (100)	5 (50)
*S. epidermidis* (n=2)	2 (100)	2 (100)	2 (100)	0	2 (100)	2 (100)	2 (100)	2 (100)
*S. pneumoniae* (n=1)	1 (100)	1 (100)	1 (100)	0	0	1 (100)	1 (100)	1 (100)

Amp=Ampicillin, Oxa=Oxacillin, Gen=Gentamicin, Van=Vancomycin, Ery=Erythromycin, Cot=Cotrimoxazole, Ceft=Ceftazidime, Cip=Ciprofloxacin

**Table 7:** % Resistance of Gram negative bacteria isolates in two primary-care hospitals in Osogbo, Nigeria

Isolates	Antibiotics (%)					
	
	Amp	Gen	Tet	Cot	Ceft	Cip
*Klebsiella* spp (n=18)	18 (100)	12 (66.7)	18 (100)	18 (100)	18 (100)	13 (72.2)
*Pseudomonas* sp (n=12)	12 (100)	12 (100)	12 (100)	12 (100)	0	5 (41.7)
*Escherichia coli* (n=7)	7 (100)	5 (71.4)	5 (71.4)	7 (100)	7 (100)	1 (14.3)
*Serratia* spp (n=1)	1 (100)	1 (100)	1 (100)	1 (100)	1 (100)	1 (100)

Amp=Ampicillin, Gen=Gentamicin, Tet=Tetracycline, Cot=Cotrimoxazole, Ceft=Ceftazidime, Cip=Ciprofloxacin

## Discussion

Before now in our environment, enterococci have been largely regarded as commensal flora and generally disregarded when isolated from clinical specimens such as wound and urine. However, there are increasing reports that this opportunistic bacterium can become pathogenic when it colonizes ecological niche where it is not normally found with potential to become invasive. Since the beginning of the 21^st^ century in the United States of America, enterococci have become major reservoir of antibiotic-resistant genes and VRE a major cause of nosocomial infections especially of the bloodstream, urinary tract and surgical sites (Cetinkaya *et al*, 2000). Also recently in our teaching and specialist hospitals, enterococci especially *E. faecalis* are increasingly isolated in pure cultures from patients with clinical evidence of infections (Taiwo *et al*, 2008; Fadiora *et al*, 2009). The aim of this study was to determine the prevalence of hospital-acquired enterococci infections in primary health care institutions in our environment.

This study recorded hospital-acquired infection rate of 22.5% for the two hospitals. This rate is higher than what is reported in developed countries with rates of 5–10% (Meers *et al*, 1980; Moro *et al*, 1986; Mayon-White *et al*, 1988; Scheel & Stormark, 1999) and also higher than rates reported from hospitals in developing countries such as Ghana (Newman, 2009) with 6.7% and Ethiopia (Gedebou, 1988) with 17%. There is need to strengthen infection control activities in Nigerian hospitals in order reduce the prevalence, mortality, morbidity, and cost of care associated with HAI.

The prevalence rate of 5.9% for hospital-acquired enterococci infection recorded in this study is considered high in view of the fact that all the isolates were from clinically infected patients over a period of just 6 months. There is need for clinicians in our environment to be aware of the role enterococci plays in clinical infections especially of urinary tract and surgical site infections. Most of the hospitalized patients (79.7%) in the two hospitals were in the age group 20–50 years and all the enterococci infections occurred in this age group. Because this age group constitutes the work force of any society, it becomes imperative to be critically aware of danger of enterococci infections and the need for prompt diagnosis, treatment and prevention.

Identification of *Enterococcus* species in this study was done using the conventional physiologic test scheme described by Facklam & Collins (1989). This scheme has been shown to correlate well with miniaturized-dehydrated tests and DNA hybridization techniques of identifying enterococci (Facklam *et al*, 1989). The scheme allowed identification of two *Enterococcus* species in this study; 85.7% *E. faecalis* and 14.3% *E. faecium* which agrees with the trend reported worldwide where *E. faecalis* is said to be responsible for about 80 to 90% of all enterococcal infections and *E. faecium* accounts for most of the others (Gordon *et al*, 1992; Facklam *et al*, 2002). This scheme is appropriate for identification of enterococci in resource limited countries such as ours as it allowed the identification of *E. faecium*, the specie that has not before been reported in Nigeria from previous studies (Iregbu *et al*, 2002; Taiwo *et al*, 2002; Taiwo *et al* 2008; Fadiora *et al*; 2009).

The enterococci isolates were resistant to multiple antibiotics with 100% of the isolates resistant to ampicillin, oxacillin, ceftazidime and gentamicin; 71.4% resistant to cotrimoxazole and 42.9% resistant to erythromycin, ciprofloxacin and vancomycin. Three isolates including the only *E. faecium* isolate were resistant to all tested antibiotics including vancomycin. Some enterococci are known to be intrinsically resistant to β-lactam antibiotics as well as many aminoglycosides while some are known to have acquired multidrug resistance to tetracycline, erythromycin, chloramphenicol and fluoroquinolones. A previous Nigerian study (Iregbu *et al*, 2002) reported 100% susceptibility of *E. faecalis* to ampicillin and vancomycin but exhibited 11% and 34% high level resistance (HLR) to gentamicin and streptomycin respectively. This present study revealed resistance rate of 100% to ampicillin and gentamicin and 43% to vancomycin. VRE may have gradually emerged in Nigeria at the turn of the century.

The emergence of VRE strains at the turn of the 20^th^ century has generated major concern among clinicians (Cetinkaya *et al*, 2000) particularly in the last two decades; virtually these strains have emerged in nosocomial infections of hospitalized patients in the USA. In this study, VRE form about 43% of all the enterococci isolates, a figure that is high when one considers the fact that vancomycin is not available for clinical use in Nigeria. The VRE isolates include two *E. faecalis* and the only *E. faecium* isolate and were resistant to all the eight antibiotics tested. This observation agrees with a recent study on wound infections in the teaching hospital in Osogbo, Nigeria which reported two *E. faecalis* isolates to be resistant to all antibiotics tested in that study (Fadiora *et al*, 2009). The clinical implication is that VRE in Nigeria may soon become a great threat unless proper control measures are initiated.

A major problem is the fact that enterococci harbor transferable genetic elements, which have an unusually broad host range and can be transferred to both Gram-negative and Gram-positive bacteria species by conjugation systems involving plasmids and transposons (Clewell and Dunny, 2002). The danger of such transmission to bacteria such as *S. aureus* and the enterobacteriaceae in our environment is apparent as some Nigerian studies have reported vancomycin resistance among clinical *S. aureus* isolates (Olayinka *et al*, 2005, Onolitola *et al*, 2007) without previous exposure to vancomycin. A cursory look at our study revealed that 50% and 20% of the *S. aureus* isolates are oxacillin and vancomycin resistant respectively. Transfer of vancomycin resistance (*van*) genes has been specifically reported in patients co-colonized with VRE and MRSA (Furuno *et al*, 2005). One limiting factor in our study is that we could not perform molecular analysis to detect the genes responsible for vancomycin resistance because of lack of facilities. Nevertheless, we believe that hospital acquired infections with VRE will become a significant health problem in this era of sophisticated medical and surgical procedures unless strategy of systematic surveillance and infection control is put in place.

Other Gram positive bacteria (*S. aureus, S. epidermidis* and *S. pneumoniae*) isolated from the patients demonstrated multiple resistances to all antibiotics except vancomycin. The Gram negative isolates (*Klebsiella* spp, *Pseudomonas* spp, *E. coli* and *Serratia* spp) were also multiply resistant and only ciprofloxacin appeared effective. These findings are in agreement with reports of recent studies in our environment on bacterial isolates of hospital environment (Taiwo *et al*, 2006), catheter associated urinary tract infection (Taiwo & Aderounmu, 2006), ear infection (Tobih *et al*, 2006), blood stream infections (Taiwo *et al*, 2008) and superficial wound infections (Fadiora *et al*, 2009). This high antibiotic resistance situation has arisen from poor antibiotic prescription policy and guidelines, with over-the-counter availability of most antibiotics including the fluoroquinolones in Nigeria. The need to regulate antibiotic consumption, prescription and usage is highly imperative in Nigeria. Multicentre studies are necessary to determine the national prevalence of enterococci infections in Nigeria and to study the evolution of vancomycin resistance strains, their distribution and spread in the country using molecular method.

## Figures and Tables

**Table 1:** Gender and age group distributions of hospitalized patients in two primary-care hospitals in Osogbo, Nigeria

	Gender		
Age Group(Years)	Male	Female	Total (%)
≤ 10	_	_	_
10–19	5	6	11 (9.3)
20–29	18	32	50 (42.4)
30–39	9	19	28 (23.7)
40–49	10	6	16 (13.6)
50–59	2	4	6 (5.1)
60–69	1	1	2 (1.7)
70–79	3	0	3 (2.5)
80–89	0	1	1 (0.8)
90–99	1	0	1 (0.8)
> 100	_	_	_
**Total**	**49 (41.5)**	**69 (58.5)**	**118 (100)**

## References

[1] Arias CA, Murray BE (2008). Emergence and management of drug-resistant enterococcal infections. Expert Rev Anti Infect Ther.

[2] Bauer AW, Kirby WWM, Sherris JC, Turk M (1966). Antibiotic susceptibility testing by standardized single disc method. Am J Clin Pathol.

[3] Cetinkaya Y, Falk P, Mayhall CG (2000). Vancomycin-resistant enterococci. Clin Microbiol Rev.

[4] Cheesbrough M (2000). Medical laboratory manual for tropical countries. Microbiology.

[5] Clewell DB, Dunny GM, Gilmore MS (2002). Conjugation and genetic exchange in enterococci. The enterococci: pathogenesis, molecular biology, and antibiotic resistance.

[6] Clinical and Laboratory Standards Institute (2007). Performance standards for antimicrobial disk susceptibility tests CLSI document M100-S17.

[7] Facklam RR, Carvalho MS, Teixeira LM, Gilmore MS, Clewell DB, Courvalin P, Dunny GM, Murray BE, Rice LB (2002). History, taxonomy, biochemical characteristics and antibiotic susceptibility testing of enterococci. The enterococci, pathogenesis, molecular biology and antibiotic resistance.

[8] Facklam RR, Collins MD (1989). Identification of *Enterococcus* species isolated from human infections by a conventional test scheme. J Clin Microbiol.

[9] Facklam R, Hollis D, Collins MD (1989). Identification of gram positive coccal and bacillary vancomycin-resistant bacteria. J Clin Microbiol.

[10] Fadiora SO, Odebunmi JF, Oladipo OJ, Taiwo SS (2009). Comparative bacteriology of superficial wound infection in a secondary and a tertiary health institution in Osogbo, Southwest Nigeria. Int J Med Med Sci.

[11] Furuno JP, Perencevich EN, Johnson JA, Wright M, McGregor JC, Glenn Morris J, Strauss SM, Roghman M, Nemoy LL, Standiford HC, Hebden JN, Harris AD (2005). Methicillin-resistant *Staphylococcus aureus* and Vancomycin-resistant Enterococci Co-colonization. Emerg Infect Dis.

[12] Gedebou M, Habte-Gabr E, Kronvall G, Yoseph S (1988). Hospital-acquired infections among obstetrics and gynaecological patients at Tikur Anbessa Hospital, Addis Ababa. J Hosp Infect.

[13] Gordon S, Swenson JM, Hill BC, Piggot NE, Facklam RR, Cooksey RC, Thornsberry C, Jarvis WR, Tenover FC (1992). Antimicrobial susceptibility patterns of common and unusual species of enterococci causing infections in the United States. J Clin Microbiol.

[14] Iregbu KC, Ogunsola FT, Odugbemi TO (2002). Susceptibility profile of *Enterococcus faecalis* isolated at the Lagos University Teaching Hospital, Nigeria. Niger Postgrad Med J.

[15] Lewis CM, Zervos MJ (1990). Clinical manifestations of enterococcal infection. Eur J Clin Microbiol Infect Dis.

[16] Mayon-White RT, Ducel G, Kereselidze T, Tikomirov E (1988). An international survey of the prevalence of hospital acquired infection. J Hosp Infect.

[17] Meers PD, Ayliffe GAJ, Emmerson AM, Leigh DA, Mayon-White RT, Mackintosh CA (1981). Report on the national survey of infection in hospitals, 1980. J Hosp Infect.

[18] Moro ML, Stazi MA, Marasca G, Greco D, Zampieri A (1986). National prevalence of hospital acquired infection in Italy 1983. J Hosp Infect.

[19] Murray BE (1990). The life and times of the enterococcus. Clin Microbiol Rev.

[20] Newman MJ (2009). Nosocomial and community-acquired infection in Korle Bu Teaching Hospital, Ghana. West Afr J Med.

[21] Olayinka BO, Olayinka AT, Onaolapo JA, Olurinola PF (2005). Pattern of resistance to vancomycin and other antimicrobial agents in staphylococcal isolates in a University Teaching Hospital. Afr J Clin Exper Microbiol.

[22] Onolitola OS, Olayinka BO, Salawu MJ, Yakubu SE (2007). Nasal carriage of methicillin-resistant *Staphylococcus aureus* with reduced vancomycin susceptibility (MRSA-RVS) by healthy adults in Zaria, Nigeria. J Trop Microbiol Biotechnol.

[23] Patterson JE, Sweeney AH, Simms M, Carley N, Mangi R, Sabetta J, Lyons RW (1995). Analysis of 110 series enterococcal infections. Medicine.

[24] Scheel O, Stormark M (1999). National prevalence of hospital acquired infection in Norway. J Hosp Infect.

[25] Taiwo SS, Okesina AB, Onile BA (2002). Invitro antimicrobial susceptibility pattern of bacterial isolates from wound infection in University of Ilorin Teaching Hospital. Afr J Clin Exper Microbiol.

[26] Taiwo SS, Aderounmu AOA (2006). Catheter associated urinary tract infection: aetiology and antimicrobial susceptibility pattern of microbial pathogens in Ladoke Akintola University Teaching Hospital. Afr J Biomed Res.

[27] Taiwo SS, Fadiora SO, Amure JO, Hassan WO, Ashiru JO (2006). Environmental reservoirs of microbial pathogens in a University Teaching Hospital in Southwestern Nigeria. J Niger Infect Contr Assoc.

[28] Taiwo SS, Fadiora SO, Fayemiwo SA (2008). High antimicrobial resistance among bacterial isolates of blood stream infections (BSI) in a Nigerian University Teaching Hospital. World J Microbiol Biotechnol.

[29] Tobih JE, Taiwo SS, Olowe OA, Olaosun OA, Adejumo SD (2006). Clinical and microbiological profiles of ear infections in Osogbo, Nigeria. Trop Doct.

